# Clinical and Molecular Advances on the Black Yeast *Exophiala dermatitidis*

**DOI:** 10.3390/ijms26146804

**Published:** 2025-07-16

**Authors:** Jakub Suchodolski, Mateusz Parol, Karolina Pawlak, Agata Piecuch, Rafał Ogórek

**Affiliations:** 1 Department of Mycology and Genetics, Faculty of Biological Sciences, University of Wrocław, Przybyszewskiego 63-77, 51-148 Wrocław, Poland; 322925@uwr.edu.pl (M.P.); karolina.pawlak@umw.edu.pl (K.P.); agata.piecuch@uwr.edu.pl (A.P.), rafal.ogorek@uwr.edu.pl (R.O.); 2 Department of Pharmaceutical Microbiology and Parasitology, Faculty of Pharmacy, Medical University of Silesian Piasts in Wrocław, Borowska 211, 50-556 Wrocław, Poland

**Keywords:** *Exophiala dermatitidis*, black yeast, melanin, cystic fibrosis, CARD9 deficiency, antifungal resistance, virulence factors

## Abstract

*Exophiala dermatitidis* is an emerging black yeast recognized in both superficial and life-threatening infections, including those in immunocompetent hosts. This narrative review focuses on recent developments (mostly between 2019 and 2025) in two major areas. First, we examined the clinical and epidemiological background of *E. dermatitidis*, with particular focus on its involvement in cystic fibrosis and CARD9 deficiency, as well as central nervous system, ocular, and systemic infections. Second, we address the molecular basis of its pathogenicity, with particular attention to melanin production, capsule formation, and metabolic adaptation. We also discuss diagnostic challenges and antifungal susceptibility, highlighting gaps between laboratory studies and clinical outcomes.

## 1. Introduction

**Figure 1:**
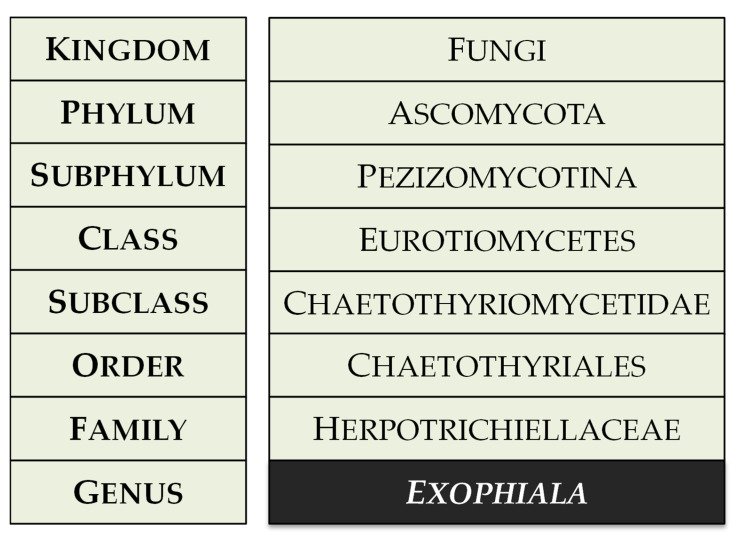
Taxonomic classification of the genus *Exophiala* according to current fungal systematics (MycoBank, accessed on 29 May 2025).

The genus *Exophiala* was described in 1966 by J.W. Carmichael, with *Exophiala salmonis* designated as the type species [1]. *Exophiala* species belong to black yeast-like fungi (BYF) and are classified within the family Herpotrichiellaceae, order Chaetothyriales, class Eurotiomycetes, phylum Ascomycota (Figure 1).

According to MycoBank.org, over 90 species of the genus *Exophiala* have been identified to date, including 35 newly described species between 2020 and May 2025. Several members of this genus are recognized as human pathogens; however, *Exophiala dermatitidis* is the most frequently isolated species worldwide, highlighting its medical importance (for more information, see Appendix A.1) [2,3,4,5,6,7,8,9].

**Figure 2:**
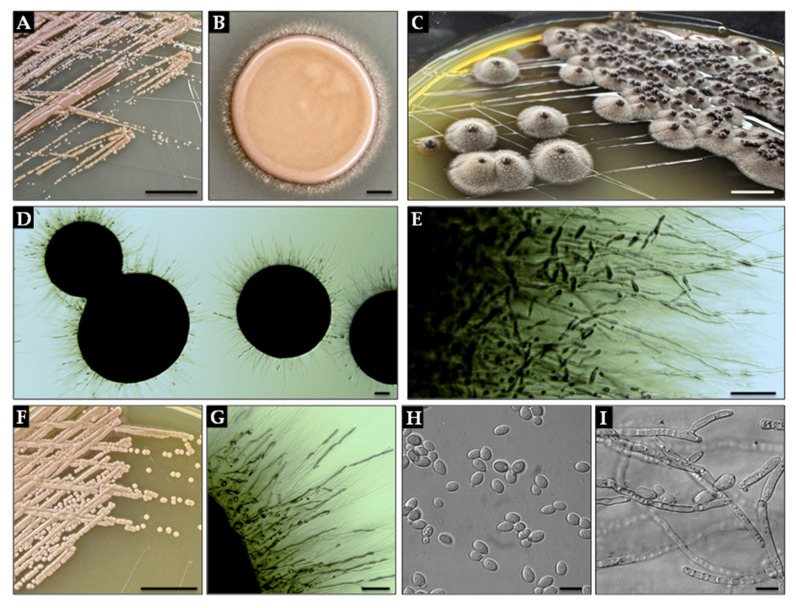
Macro- and microscopic morphology of *Exophiala dermatitidis*. (**A**) Colony morphology on YPD agar after 3 days at 24 ± 2 °C, showing a moist, yeast-like texture with a glossy surface and defined margins (scale bar = 10 mm). (**B**) Colony formed by spot-inoculation of 5 µL cell suspension on YPD agar after 3 days at 24 ± 2 °C (scale bar = 1 mm). (**C**) Mature colony on YPD agar after 7 days at 24 ± 2 °C, displaying a suede-like, domed surface with gray aerial hyphae and black pigmentation (scale bar = 5 mm). (**D**) Enlarged view of colonies from panel (**A**) under transmitted light, revealing filamentous margins with radiating hyphae (scale bar = 100 µm). (**E**) Conidiogenesis at colony edge, showing tapering annellides with ellipsoid conidia (scale bar = 100 µm). (**F**) Colony growth on YPD agar after 3 days at 37 ± 2 °C, with markedly reduced aerial hyphal development and predominance of yeast-like morphology (scale bar = 10 mm). (**G**) Close-up of colony margin from panel (**F**), illustrating sparse hyphal elements and budding cells (scale bar = 100 µm). (**H**) Blastoconidia observed in early culture under DIC (scale bar = 10 µm). (**I**) Mature septate hyphae and annellidic conidiogenous cells from panel (**C**) visualized with DIC (scale bar = 10 µm). For more morphological details, see Appendix A.2. All images represent isolate *E. dermatitidis* 7U2 (isolated by A. Piecuch, unpublished). Panels (**A**–**C**,**F**) photographed by M. Parol; panels (**D**,**E**,**G**–**I**) are by J. Suchodolski.

Morphologically, *E. dermatitidis* exhibits facultative dimorphism—forming yeast-like blastoconidia at 37 °C and filamentous hyphae at lower temperatures (Figure 2). This fungal species also displays a polyextremotolerant phenotype, thriving across a temperature range of 4 °C to 47 °C, growing in media containing up to 20% NaCl, and surviving in highly acidic to alkaline conditions (pH 2.0–12.0) [10].

Over the past two decades, a growing number of case reports have highlighted its pathogenic potential (reviewed in [2]). The fungus is frequently isolated from individuals with structural lung disease or immunological defects, including cystic fibrosis, hematological malignancies, CARD9 deficiency, or those receiving immunosuppressive therapy [11,12,13,14,15,16,17]. Recent evidence has also associated *E. dermatitidis* with conditions such as Crohn’s disease, as well as central nervous system, ocular, and scalp infections. *E. dermatitidis* has also emerged as an etiological agent in polymicrobial infections, where it has been co-isolated with other yeast-like and filamentous fungi, bacteria, or parasitic nematodes.

*E. dermatitidis* displays a multi-path virulence strategy that includes melanin production, capsule formation, high-affinity nutrient uptake systems, and phenotypic plasticity [2]. However, the strain-dependent variability between clinical and environmental isolates complicates diagnosis and treatment.

While earlier reviews—most notably by Kirchhoff et al. [2]—provided important foundational knowledge, substantial advances have since been made in the genomics of *E. dermatitidis*, as well as in our understanding of its clinical outcomes and virulence traits. Thus, this narrative review synthesizes recent developments in two main areas: (I) the clinical and epidemiological aspects of *E. dermatitidis* infections, and (II) the molecular mechanisms underlying its pathogenicity. We also summarize the diagnostic approaches and antifungal susceptibility patterns, with an emphasis on the gaps between laboratory findings and real-world treatment outcomes.

## 2. Study Selection

The literature was searched using Google Scholar for publications between 2019 and May 2025, with the keywords: “*Exophiala dermatitidis*”, “melanin”, “infection”, and “virulence”. The initial search yielded approximately 2000 records. Each entry was screened to verify that it represented a primary scientific publication, book chapter, or thesis, rather than a citation, duplicate, or unrelated result. After this initial validation, 172 publications addressing *Exophiala dermatitidis* were retained for full-text analysis. Of these, 123 were selected as directly relevant and were used in the development of this review. This final set includes post-2019 studies, as well as a limited number of pre-2019 publications that were selectively included to provide background or historical context.

## 3. Ecological Background of *Exophiala dermatitidis*

Due to polyextremotolerance, *Exophiala* spp. are widely distributed in diverse environments, particularly in warm, humid, and hydrocarbon-rich habitats such as decaying wood, organic debris-laden soils, and aquatic biofilms [2,18,19,20].

### 3.1. Occurrence in Human Environment

*E. dermatitidis* has long been recognized as a colonizer of human-made environments such as dishwashers, washing machine seals, bathrooms, and saunas, where conditions of heat and moisture support its growth [2,18,21,22,23].

In recent years, however, a few new reports have expanded the known ecological range of this fungus. It has been detected in the human oral cavity during routine screening, suggesting an ability to colonize mucosal surfaces beyond its typical niches [24]. Moreover, *Exophiala* spp. have been reported as a frequent component of the human skin microbiome, typically co-existing asymptomatically in immunocompetent individuals [25].

Notably, *E. dermatitidis* has also been isolated from stored apples in marketplaces, suggesting an ecological interface between anthropogenic and natural habitats [26].

### 3.2. Natural Habitat, Origin and Hypothetical Transmission Route

It has been hypothesized that original niche of *Exophiala dermatitidis* was in tropical rainforests, particularly on the surfaces of wild fruits [20]. Its dispersal has been attributed to frugivorous animals such as bats and birds acting as passive vectors via fecal excretion [27]. A three-year field survey involving over 3000 environmental samples found no evidence of soil- or plant-based transmission, supporting the fruit-vector hypothesis [2,20].

Notably, a 2022 study have identified *Exophiala* spp. colonizing the upper respiratory tract (choanae and trachea) of psittaciform birds—including *Amazona*, *Ara*, *Cacatua*, *Eclectus*, and *Psittacus* species—marking the first ecological link between frugivorous hosts and airway colonization [28]. Colonization appeared asymptomatic, suggesting that these birds may serve as transient reservoirs or contribute to fungal dispersal through inhalation and excretion pathways [28].

**Figure 3:**
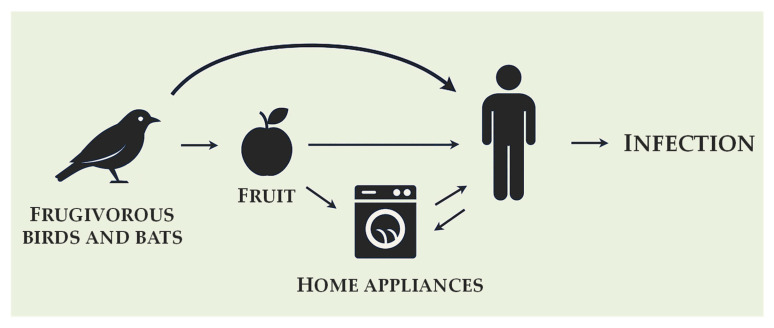
Hypothesized ecological transmission pathway of *Exophiala dermatitidis* from natural reservoirs to human-associated niches via frugivorous vectors. Arrows indicate the proposed direction of transmission (original figure created by the authors).

The natural reservoirs and transmission routes of *E. dermatitidis* remain unclear. However, its presence in wild fruits, frugivorous birds, and human environments suggests a possible ecological link. Based on the reviewed literature, we propose a hypothetical transmission pathway in Figure 3.

## 4. Clinical and Epidemiological Aspects of *Exophiala dermatitidis*

Earlier works highlighted the association of *E. dermatitidis* with both superficial and invasive infections, especially in patients with immune or anatomical vulnerabilities, with higher incidence reported in East Asia [2]. However, the true epidemiological profile remains unclear due to underreporting and diagnostic limitations. Rare pathogens or natural inhabitants of human skin may be omitted during the diagnostic process and ruled as contamination [29,30]. Anecdotal evidence suggests that *E. dermatitidis* isolates—particularly from respiratory specimens—are often treated as an environmental contaminant and discarded without further investigation. Although this practice regarding *Exophiala* species has not been formally documented in the literature, it may contribute to an underestimation of its clinical relevance and prevalence. Nevertheless, recent studies have expanded our understanding of the clinical spectrum of *E. dermatitidis*, its host susceptibility, and emerging immune-related associations.

### 4.1. Predisposing Factors

A range of predisposing conditions have been associated with *E. dermatitidis* infections, including older age, female sex, cancer, liver or pancreatic insufficiency, and catheter use. These associations—primarily based on case reports and observational studies—have been reviewed in the earlier literature [11,12,14,15,16,17].

However, two conditions have consistently stood out due to the strength and reproducibility of the supporting evidence: cystic fibrosis (CF) and CARD9 deficiency. Recent molecular and genomic studies have further elucidated how *E. dermatitidis* adapts to these host environments.

#### 4.1.1. Cystic Fibrosis

CF is a genetic disorder caused by mutations in cystic fibrosis transmembrane regulator (CFTR) gene [31]. Among the many effects of CF, one is the production of viscous mucus, which is utilized by bacteria (e.g., *Staphylococcus aureus*, *Pseudomonas aeruginosa*) and fungi (e.g., *Candida albicans*, *Aspergillus fumigatus*) to establish infection [32]. The first mentions of *E. dermatitidis* isolated from a CF patient originate from the same individual, a 5-year-old and then a 7-year-old girl, and were reported in 1990 and 1992 [33,34]. Since then, *E. dermatitidis* has been isolated numerous times from CF patients, highlighting its ability to exploit this condition [11,12,13]. Also, the colonization of *Aspergillus* spp. and *Candida* spp. was observed to be a predisposing factor in *Exophiala* spp. infections in CF patients [11,12,14].

Recently, Kurbessoian et al. [32] reported the acquisition of genetic mutations in *E. dermatitidis* isolates obtained from a single CF patient, with samples collected 2 years apart. One of the most notable examples are mutations (SNP and INDEL) in Ap-3 complex subunit delta, DNA repair protein Rad50, and a regulator of nonsense transcripts 1-like protein (HMPREF1120_06837) [32]. The various mutations reported in CF *E. dermatitidis* isolates may indicate the process of adaptation to this specific environment.

For example, some isolates of *E. dermatitidis* were observed to produce hyper-filamentous phenotype, which could be a result of mutations found in *RAD50* [32]. In *Saccharomyces cerevisiae*, *RAD50* is a member of the Rad52 epistasis group, whose genes are involved in the repair of double-stranded breaks in DNA [35].

Iron acquisition might also be a possible virulence factor of *E. dermatitidis* in CF patients [32]. Mutations in *MRS4*, encoding the fungal mitochondrial iron transporter, often resulting in the loss of its function, have been described as potentially beneficial during chronic CF lung infections, with studies on *Candida lusitaniae* and *E. dermatitidis* isolates [36]. In response to the loss of Mrs4p function, the observed enhanced expression of the siderophore acquisition pathways and surface ferric reductases may enable cells to accumulate high levels of iron while limiting its accumulation in the mitochondria, which protects the mitochondria from damage [36]. The increased iron levels could support cell survival in iron-limited environments, making it a crucial factor in microbial competition [36].

#### 4.1.2. CARD9 Deficiency

Caspase recruitment protein domain 9 (CARD9) is one of the key proteins that mediates signaling pathways involved in both innate and adaptive immune responses to various pathogens [37]. *CARD9* gene is expressed in almost all organ systems, primarily in myeloid cells, but also in cardiomyocytes and endothelial cells [37,38]. CARD9 mediates signals from pattern recognition receptors (PRRs), including Toll-like receptors (TLRs) and C-type lectin receptors (CLRs), which, in turn, activate signaling pathways such as NF-κB and MAPK, which regulate inflammatory responses [38]. Additionally, CARD9 plays a key role in adaptive immunity by promoting the differentiation of T_H_ cell subsets, particularly T_H_17 and T_H_22 [38,39]. In response to the signaling pathways activated by CARD9, immune cells (neutrophils, macrophages, and dendritic cells) secrete cytokines (e.g., TNF-α, IL-6, IL-17 and IL-1β) and chemokines (e.g., CXCL1, CXCL2, and CXCL8) [37,38,39]. Mutations in *CARD9* resulting in loss of function (e.g., premature stop codon in the coding sequence) have been observed to increase susceptibility to specific fungal infections by impairing T_H_17 cell differentiation, leading to cytokine deficiency and making it a significant risk factor [39,40,41].

Fungal species identified in CARD9-deficiency patients include *C. albicans*, *A. fumigatus*, *Cryptococcus neoformans*, *Malassezia furfur*, and *Trichophyton* spp., but also *E. dermatitidis* [42,43,44,45]. Infections in CARD9-deficient patients are generally considered to be caused by a single fungal species, as is the case with *E. dermatitidis* [42,46].

### 4.2. Recent Clinical Outcomes

Beyond host-related predispositions, a growing body of literature documents increasingly diverse clinical manifestations of *E. dermatitidis*. These span localized infections to severe systemic and neuroinvasive disease. Major recent presentations are outlined in the following chapters. For clarity, these outcomes are also summarized in Table 1.

#### 4.2.1. Pulmonary Infections Beyond Cystic Fibrosis

Recent reports confirm the involvement of *E. dermatitidis* in pulmonary infections among non-CF immunocompromised patients, including individuals undergoing organ transplantation or chemotherapy, or those living with HIV/AIDS [47,48].

In one fatal post-transplant case, *E. dermatitidis* caused invasive infection originating from a thoracotomy wound and extending into the pleural cavity and lung allograft [47]. In a multicenter study, *E. dermatitidis* and *Exophiala phaeomuriformis* were isolated from bronchoalveolar lavage samples of immunosuppressed patients [48].

#### 4.2.2. Central Nervous System Infections

CNS infections caused by *E. dermatitidis* represent the most severe and fatal form of phaeohyphomycosis. As of 2021, 22 cases of confirmed meningoencephalitis have been reported, with 17 occurring in patients without known predisposing factors—all of whom either relapsed or died despite antifungal therapy, suggesting a possible genetic susceptibility such as CARD9 deficiency [43,49,68].

Most immunocompetent CNS cases occurred in East or South Asia, aligning with previously noted regional patterns [2,49]. Nearly half of cases occurred under the age of 20, with the youngest being a 3-year-old from China [49]. This demographic pattern suggests a genetic susceptibility, possibly tied to CARD9 deficiency reported in Asian patients with severe fungal infections [49].

Recent experimental data have further reinforced the neurotropic character of *E. dermatitidis*. In vitro studies on SH-SY5Y neuroblastoma cells demonstrated that melanized extracellular vesicles (EVs) from *E. dermatitidis* caused severe cytotoxicity, while direct pseudohyphal invasion lead to rapid neuronal destruction within 72 h [50]. *E. dermatitidis* has also been shown to utilize neurotransmitters like GABA and acetylcholine as sole carbon sources tissues (see Section 5.4. for further information), suggesting metabolic adaptation to neural tissues [50].

#### 4.2.3. Fungemia and Disseminated Infections

Fungemia and systemic disease in immunocompromised patients due to *E. dermatitidis* are rare but clinically significant due to diagnostic delays, antifungal resistance, and overlap with common pathogens.

A review by Tzar et al. [51] identified nine fungemia cases (between 2017 and 2021), including a healthcare-associated outbreak and multiple reports from Asia and South America. Most patients had underlying malignancies or were catheterized. Delayed treatment was associated with poor outcomes. Additional cases linked fungemia to corticosteroid use in chronic obstructive pulmonary disease (COPD) [52], highlighting immune suppression as a key risk factor.

Disseminated infection was reported by Hagiya et al. [53] in a post-transplant patient, with histologically confirmed spread to multiple organs despite antifungal therapy, demonstrating the pathogen’s invasive potential and treatment limitations. Nakatani et al. [69] reported a case of a neutropenic infant with disseminated *E. dermatitidis* infection, successfully managed with a combination of antifungal therapy and surgery.

Moreover, *E. dermatitidis* was found to be enriched in the gut mycobiome of patients with cardiovascular disease [70], suggesting possible systemic translocation in vulnerable hosts.

#### 4.2.4. Association with Crohn’s Disease

Species of the genus *Exophiala* have been linked to Crohn’s disease (CD) in both clinical case reports and molecular studies. Naik et al. [54] described the first case of non-invasive colonic phaeohyphomycosis caused by *Exophiala oligosperma* in a CD patient, which was successfully treated with voriconazole. Another report documented an ocular infection with *E. dermatitidis* in a woman with CD, suggesting that host immune dysregulation may facilitate opportunistic colonization [55].

A 2022 observational study employing ITS-based fungal community profiling found that *E. dermatitidis* was significantly enriched in patients with active CD, particularly in those with non-B1 subtypes associated with intestinal stenosis or fistulizing disease [56]. Positive correlations were also observed between fungal abundance, elevated platelet counts, and disease severity indicators [56].

More recently, functional studies in a 2025 murine colitis model relevant to CD demonstrated that *E. dermatitidis* exacerbates intestinal inflammation via the Mincle–Syk–CARD9–NF-κB pathway, promoting CX3CR1^+^ macrophage activation [45]. Reduced inflammation in CARD9-deficient mice underscores the immunological basis of this interaction and supports a mechanistic link between *E. dermatitidis* and CD pathogenesis [45].

#### 4.2.5. Ocular Infections

Although rare, ocular infections by *Exophiala* spp. pose serious risks, particularly following eye surgeries. Recent reports describe *E. dermatitidis* keratitis and endophthalmitis linked to procedures like keratoplasty or cataract surgery [57,58,59].

In two keratitis cases, *E. dermatitidis* was isolated post-keratoplasty, with outcomes including graft failure and vision loss [58,59]. Corticosteroid use was noted as a risk factor. Another case involved delayed-onset endophthalmitis after cataract surgery in a diabetic patient [57]. Interestingly, intraocular infections represent only ~6.8% of all *Exophiala* infection, but only eight cases of *Exophiala*-related endophthalmitis have been documented as for 2022 study, three of which were caused by *E. dermatitidis* [57].

#### 4.2.6. Scalp Infections and Hair Loss Involving *Exophiala dermatitidis*

Although not a primary cause of alopecia, *E. dermatitidis* has recently been isolated from affected scalps. In a study of school-aged girls with hair loss in Iraq, it was detected as a minor component of the scalp mycobiota, alongside more common species like *Epidermophyton floccosum* and *Cladophialophora carrionii* [60]. Its presence may reflect opportunistic colonization in compromised or dysbiotic scalp environments, but its role in pathogenesis remains unclear. A metagenomic analysis of skin mycobiome (with scalps being one of the samples) revealed an abundance of *Exophiala* species (including *E. dermatitidis*) that were also observed in healthy individuals [25].

#### 4.2.7. *Exophiala dermatitidis* in Polymicrobial Infections

*E. dermatitidis* has recently been implicated in polymicrobial infections. Miyoshi et al. [61] described a pulmonary co-infection involving this fungal species and *Mycobacterium avium* in a patient with bronchiectasis, highlighting the role of local anatomical changes rather than systemic immunosuppression.

Ahamad et al. [62] reported a bloodstream co-infection with *E. dermatitidis* and *Mycobacterium canariasense* in a patient with a central venous catheter, underscoring the pathogen’s biofilm-forming capacity on indwelling devices. Kohli et al. [63] described a respiratory co-infection involving *C. neoformans*, where *E. dermatitidis* may have worsened clinical severity despite not being the primary agent.

Salvador et al. [64] documented a mixed onychomycosis involving *E. dermatitidis* and *Candida parapsilosis*, both capable of forming biofilms. Notably, *C. parapsilosis* dominated within the mixed biofilm, suggesting competitive or synergistic microbial interactions.

Kirchhoff et al. [65] investigated *E. dermatitidis* interactions with *P. aeruginosa* under cystic fibrosis-like conditions. Initially, *P. aeruginosa* enhanced fungal biofilm development, but later-stage inhibition was mediated by quorum-sensing molecules. The bacterium also induced a morphological shift toward conidial forms, potentially impacting fungal virulence. In vivo studies confirmed cooperative pathogenicity, as filtrates from *P. aeruginosa* biofilms increased the virulence of *E. dermatitidis*.

Experimental findings by Quan [66] revealed that *E. dermatitidis* may be outcompeted by faster-growing fungi like *A. fumigatus* under nutrient-rich conditions, but persists in selective or extreme environments, indicating niche-specific competitive dynamics.

A rare co-infection involving *E. dermatitidis* and the parasitic nematode *Angiostrongylus cantonensis* was reported by Ma et al. [43] in a child with CARD9 deficiency, demonstrating the compounded risk of systemic infection in genetically immunocompromised hosts.

Hatta et al. [67] described sequential infections with two *Exophiala* species in the same patient, further illustrating the genus’s clinical diversity and persistence.

A recent case by Tzar et al. [51] documented *E. dermatitidis* fungemia in a previously healthy patient with severe COVID-19 pneumonia. The infection was confirmed via the phenotypic identification of dematiaceous, flask-shaped budding yeasts from blood cultures. Despite treatment with amphotericin B and, later, itraconazole, the patient succumbed to multiorgan failure. This case underscores the potential for *E. dermatitidis* to act as a lethal opportunistic pathogen in the setting of COVID-19-related immune dysfunction, particularly when antifungal therapy is delayed or suboptimal.

#### 4.2.8. Expanding Clinical Perspectives—Animal Infections

Although *E. dermatitidis* is primarily known as a human pathogen, isolated cases in animals have been reported, typically in immunocompromised hosts or following medical interventions.

Earlier reports identified *E. dermatitidis* in the liver of fruit bats (*Eidolon helvum*), with experimental studies confirming its neurotropism in mice, and cases have been documented in companion animals, including subcutaneous and intra-abdominal infections in immunosuppressed dogs [71,72,73].

However, feline infections are newly emerging. A subcutaneous infection at the site of a feeding tube was reported by Osada et al. [74], with fatal outcome despite treatment. A second case described systemic intra-abdominal and granulomatous lesions—marking the first documented systemic phaeohyphomycosis in a cat [75].

## 5. Updated Insights into Virulence Factors of *Exophiala dermatitidis*

Given the broad clinical spectrum and high mortality in selected settings, understanding the pathogenic traits of *E. dermatitidis* is essential. Since earlier summaries [2], further mechanistic and experimental insights have refined our understanding of its virulence traits, particularly in terms of melanin-dependent immune evasion, capsule formation, and niche-specific enzymatic adaptations. Below, we update and contextualize these attributes in light of recent findings.

### 5.1. Melanin as a Virulence Factor

Melanin is a dark polymeric pigment produced as a protection against environmental stressors. In *E. dermatitidis*, it contributes to immune evasion, oxidative stress resistance, drug resistance, and colonization of the host. Recent studies demonstrate that *E. dermatitidis* encodes the genetic and enzymatic pathways necessary for at least three distinct melanin pathways—1,8-dihydroxynaphthalene (DHN)–melanin, L-3,4-dihydroxyphenylalanine (L-DOPA)–melanin, and pyomelanin (Table 2)—with DHN–melanin being the primary and functionally dominant type [76,77].

The DHN–melanin pathway, catalyzed by polyketide synthase (Pks1), is highly conserved among BYF and constitutively produced during host infection by *E. dermatitidis* [76]. In contrast, the L-DOPA and pyomelanin pathways are either less active or conditionally produced, and serve as auxiliary systems under specific stress conditions [76]. Phylogenetic analysis has recently shown that *PKS1* homologs in BYF are structurally diverse outside the conserved keto-synthase (KS) domain [76].

The melanin polymer in *E. dermatitidis* has been characterized as amorphous and heavily cross-linked (particle sizes of 20–30 nm), rich in aromatic bonds with low crystallinity, and 0resistant to acid hydrolysis or enzymatic degradation [80]. Also, melanin was described as integrated into the cell wall, forming an external protective layer and occasionally accumulating extracellularly, particularly under stress [80].

Melanin production in *E. dermatitidis* is inducible via oxidative stress (H_2_O_2_), UV radiation, and heavy metals (Cu^2+^, Fe^3+^) [78,79]. Recent transcriptomic data have shown strong upregulation of DHN–melanin pathway genes (*PKS1*, *AYG1*, *ARBA*, *ABR2*) in response to above mentioned stressors [78,79]. Additionally, it was proved that these genes often co-express with other stress response genes, such as those encoding SODs, catalases, and transporters for Cu^2+^ and Fe^3+^ [76].

In terms of pathogenicity, melanin has been shown to (I) mask pathogen-associated molecular patterns (PAMPs), reducing recognition by host immune cells; (II) neutralize reactive oxygen species (ROS) and lysosomal enzymes; and (III) promote intracellular survival in macrophages.

Knockout of *PKS1* in *E. dermatitidis* impaired its ability to invade skin in ex vivo models, highlighting the essentiality of DHN–melanin in early infection stages [77]. Wild-type strains induced epidermolysis and the keratinocyte internalization of melanin, potentially enabling a “Trojan horse” mechanism for delivering virulence factors [77].

Melanin has been shown to contribute to antifungal resistance by sequestering drugs such as amphotericin B and azoles, preventing them from reaching their intracellular targets [32]. Stress-induced upregulation of Cu^2+^, Fe^3+^ transporters and biofilm formation genes suggested melanin may act in concert with other survival strategies under antifungal pressure [79].

Beyond its cellular functions, melanin has been shown to bind cytokines and free radicals, potentially suppressing local immune responses [32]. Its role in metal chelation supports both detoxification and micronutrient acquisition [78].

### 5.2. Capsule Formation

A polysaccharide capsule is a well-characterized virulence factor in *C. neoformans*, where it mediates immune evasion by inhibiting phagocytosis, suppressing pro-inflammatory responses, and resisting complement activation [81]. Although rarely reported among other fungal species, similar capsule-like structures have been observed in *E. dermatitidis*, suggesting a convergent strategy for host immune modulation.

*E. dermatitidis* produces extracellular polysaccharides—acid mucopolysaccharides—that form a capsule-like structure enveloping yeast cells [2]. This material plays a similar role as in *C. neoformans* [2]. Also, early experimental work by Nishimura and Miyaji [82] demonstrated that these polysaccharides modulate interactions between fungal cells and host neutrophils, possibly impairing effective immune clearance.

More recent genomic and phenotypic comparisons by Song et al. [83] confirmed that *E. dermatitidis* and its close relative *Exophiala spinifera* produce this capsule-like material during exponential growth. Despite the absence of conserved, canonical capsule-associated gene clusters across different strains, capsule formation remains a shared trait among clinical isolates, especially those derived from neurotropic infections. Notably, genes uniquely expressed in brain-derived strains of *E. dermatitidis*—including those with Myb-like DNA-binding domains—suggest that encapsulation might be tightly regulated and context-dependent, particularly during dissemination or CNS invasion [83].

### 5.3. Hydrolytic and Virulence-Associated Enzymes

While earlier reviews acknowledged the enzymatic potential of *E. dermatitidis* [2], recent studies have refined our understanding of its enzymatic range. Particularly since 2019, growing experimental and transcriptomic evidence has highlighted the strain-specific and condition-dependent production of hydrolytic and redox-related enzymes. These activities may play key roles in niche adaptation, immune evasion, and tissue invasion (Table 3). The following section synthesizes updated data on enzymatic virulence traits, highlighting both established and emerging mechanisms.

#### 5.3.1. Catalase

Catalase is a heme enzyme that detoxifies hydrogen peroxide (H_2_O_2_), a reactive oxygen species produced by host immune cells, enhancing fungal resistance to oxidative stress and aiding in survival during neutrophil attacks [84]. *E. dermatitidis* exhibits high-level resistance to high concentrations of H_2_O_2_, withstanding levels of up to 6–9 mM [85]. Notably, catalase activity is consistently detected in 100% of *E. dermatitidis* strains, regardless of the year of study, strain origin (clinical or environmental), or testing conditions [86,87]. Furthermore, Song et al. [85] predicted the presence of a bifunctional catalase/peroxidase enzyme in *E. dermatitidis*, suggesting an additional mechanism for reducing H_2_O_2_ and contributing to the species’ oxidative stress resilience.

#### 5.3.2. Urease

Urease is a nickel-dependent enzyme that hydrolyzes urea into ammonia and bicarbonate, enabling fungal pathogens to neutralize acidic environments and evade macrophage-mediated killing, enhancing survival during infection [88]. While earlier studies by Sav et al. [86] reported that 100% of *E. dermatitidis* strains exhibited urease activity, subsequent research revealed variability in this trait. For example, Song et al. [85] found that only 5 out of 20 tested strains displayed urease activity, with 3 of these exhibiting weak activity. More recently, a 2024 study observed that 56% of isolates (14 out of 25 strains) were urease-positive [87], indicating that urease activity may not be as ubiquitous in this species as previously thought.

Moreover, *E. dermatitidis* demonstrates ammonia tolerance, with moderate growth on ammonium-containing media and an increase in pH during growth in maltose/asparagine media [89]. This interplay between urease activity and ammonia tolerance suggests a dual mechanism by which *E. dermatitidis* adapts to and modifies acidic microenvironments, potentially enhancing its capacity for colonization and virulence in hostile conditions. Notably, in the feline case reported by Osada et al. [74] the infection was associated with azotemia (elevated blood urea nitrogen).

#### 5.3.3. DNase

Extracellular DNase is emerging as a potential virulence factor in fungi, similar to its role in bacterial pathogens, where it aids immune evasion by degrading neutrophil extracellular traps (NETs) and other DNA-based defenses [90]. In the case of *E. dermatitidis*, DNase activity has rarely been observed. A 2016 study reported this activity in only 3 out of 144 tested strains, while no activity was detected in a 2024 study involving 25 strains, both conducted on DNase Test Agar [86,87]. However, a 2018 study using an ex vivo human skin model and RNA sequencing demonstrated that the DNase gene is actively regulated during skin infection in both wild-type and melanin-deficient strains of *E. dermatitidis* [77]. This discrepancy suggests that DNase activity may not manifest under standard in vitro conditions but could play a role in vivo during host–pathogen interactions.

#### 5.3.4. Protease

Proteases are crucial fungal virulence factors, enabling host invasion, immune evasion, and nutrient acquisition by degrading structural barriers and immune components [91]. Some also serve non-canonical roles, such as adhesion and biofilm formation, enhancing pathogenicity [91]. In the case of *E. dermatitidis*, protease activity has been observed infrequently under standard in vitro conditions. A 2016 study reported protease activity in only 4 out of 144 tested strains, while a 2017 study found this activity in just 2 out of 25 strains [85,86]. Both studies relied on conventional microbiological media, which may underestimate protease activity during infection. In contrast, a 2018 ex vivo human skin model with RNA sequencing revealed the expression of virulence-related proteases, including excreted serine proteases, in *E. dermatitidis* [77]. These findings suggest that protease gene expression is context-dependent and likely enhanced in host-like conditions, emphasizing their potential role in tissue invasion and immune modulation during infection.

#### 5.3.5. Hemolysins

Hemolysins facilitate iron acquisition by lysing red blood cells and are well-characterized virulence factors in fungi such as *C. albicans* [92]. In *E. dermatitidis*, hemolytic activity appears to be strain-dependent and variably expressed, with conflicting reports in the literature.

Initial studies by Song et al. [85] found no hemolysis across multiple strains incubated on blood agar, suggesting that hemolysin production is absent or negligible under those conditions. In contrast, de León et al. [10] observed α-hemolysis in 93% of tested strains—clinical, environmental, and indoor—at both 28 °C and 37 °C, indicating a conserved but mild hemolytic phenotype. Notably, β-hemolysis was absent in all samples.

Seneviratne et al. [92] highlighted intra-species variability, reporting β-hemolysis in one isolate and complete absence in another, both obtained from the same patient. This divergence may reflect host-specific regulation or phase variation.

The mechanistic basis of hemolysin production in *E. dermatitidis* remains undefined, and the absence of β-hemolysis in large-scale studies implies a modest contribution to virulence. Nevertheless, consistent α-hemolytic activity may assist in iron scavenging or subtle immune evasion, warranting further functional characterization.

#### 5.3.6. Other Enzymes and Knowledge Gaps

To date, several other enzyme activities have been studied in *E. dermatitidis*, including phospholipases and oxidase, all of which are commonly associated with fungal virulence [93,94]. However, none of the reported strains exhibited these enzymatic activities [86,87]. This suggests that either *E. dermatitidis* generally lacks these activities, or, as observed for DNase and protease, such activities may occur infrequently and remain undetected under current experimental conditions.

Moreover, the roles of esterases, superoxide dismutase (SOD), elastases, and hyaluronidases in *E. dermatitidis* remain unexplored, despite their known contributions to virulence in other fungi, such as immune evasion (SOD) and tissue invasion (esterases, elastases, hyaluronidases) [95,96,97]. Exploring such activities could reveal new insights into the pathogenic mechanisms of *E. dermatitidis*. Notably, during ex vivo human skin infection, a gene encoding a triacylglycerol lipase—putatively containing acetyl esterase and lipase domains—was among the most highly upregulated transcripts, suggesting a potential role for such enzymatic activity in host interaction [79].

However, some activities with a lesser role in virulence have been reported in *E. dermatitidis*. Esculin hydrolysis has been inconsistently observed, with positive activity reported only in a minority of *E. dermatitidis* strains [86,87]. While its biological significance in this species remains unclear, esculin hydrolase activity is phenotypically used in some bacteria as a surrogate marker of virulence [98,99].

### 5.4. Carbon Source Utilization and Metabolic Plasticity

The ability of fungal pathogens to utilize diverse carbon sources within the human host is increasingly recognized as a determinant of niche adaptation, and virulence [100,101,102,103]. In contrast to nutrient-rich laboratory media, host tissues provide variable and often limiting carbon environments, shaped by anatomical location, immune activity, and microbial competition [100,101]. For example, glucose availability differs substantially between blood, mucosal surfaces, and inflamed tissues, whereas alternative carbon sources such as lactate, amino acids, or neurotransmitters may dominate specific host niches [102,103].

In this context, emerging data suggest that *E. dermatitidis* exhibits comparable metabolic flexibility. Recent phenotypic and genomic analyses have confirmed that *E. dermatitidis* can metabolize a wide spectrum of carbon sources relevant to the host environment. Sav et al. [86] reported that both clinical and environmental isolates grow efficiently on substrates such as glucose, sorbitol, mannitol, and N-acetyl-D-glucosamine—a compound derived from fungal cell wall turnover. Moreover, comparative metabolic fingerprinting revealed substantial strain-level variability in carbon source utilization, highlighting the organism’s adaptability [10]. Notably, *E. dermatitidis* strains consistently oxidized over 90 tested carbon substrates, including sugars, amino acids, neurotransmitters (dopamine, serotonin, GABA, acetylcholine), and even polycyclic aromatic hydrocarbons [10,50]. Selected examples of physiologically relevant carbon sources utilized by *E. dermatitidis* are summarized in Table 4.

### 5.5. Other Virulence-Associated Traits

In addition to melanin production and enzymatic activity, *E. dermatitidis* exhibits traits that support colonization and survival in host and environmental settings. These include surface hydrophobicity, adhesion, biofilm formation, thermotolerance, and morphological plasticity—features previously described in reviews such as that of Kirchhoff et al. [2]. However, the recent literature presents little mechanistic progress in these areas.

*E. dermatitidis* remains capable of strong surface adhesion to both abiotic (e.g., plastic, glass) and biotic substrates (e.g., epithelial cells), likely facilitated by hydrophobic cell wall components. This adhesion often precedes biofilm formation, observed across clinical and environmental strains. Biofilms confer antifungal tolerance and protection from host defenses, yet their molecular regulation remains poorly defined.

The species also retains robust thermotolerance, with growth observed at 37–42 °C—a trait not shared by all *Exophiala* spp. and considered critical for systemic pathogenicity [2].

## 6. Diagnostic and Therapeutic Considerations

The effective clinical management of *E. dermatitidis* infections relies on accurate diagnosis and the timely initiation of antifungal therapy. However, challenges persist due to its slow growth, phenotypic variability, and resistance patterns. Recent developments in molecular and proteomic diagnostics, along with accumulating case data and antifungal susceptibility profiles, have refined the approach to this opportunistic pathogen. This section summarizes the evolving diagnostic landscape and therapeutic considerations based on the post-2019 literature.

### 6.1. Diagnostic Strategies

Given the diversity of available diagnostic techniques for *E. dermatitidis*, ranging from classical culture methods to advanced molecular and proteomic tools, a comparative summary is provided in Table 5.

#### 6.1.1. Classical Culture-Based Diagnostics

Culture remains a primary diagnostic method for *E. dermatitidis*, especially in resource-limited settings. Growth is typically achieved on Sabouraud dextrose agar (SDA) or malt extract agar (MEA) incubated at 35 °C for 48–72 h [57,59]. Slow growth and risk of bacterial overgrowth, particularly in polymicrobial samples, limit a culture’s sensitivity [2].

Selective media, such as PDA with Bengal rose, *Burkholderia cepacia* selective agar (BCSA), erythritol chloramphenicol agar (ECA), and Sabouraud gentamicin chloramphenicol agar (SGCA), can enhance recovery [2]. Nevertheless, morphological similarities with other black yeasts and the low fungal burden in invasive cases limit a culture’s diagnostic value [52].

#### 6.1.2. Morphological and Staining Techniques

Microscopic observation with lactophenol blue staining reveals brown-pigmented hyphae characteristic of dematiaceous fungi. However, due to overlapping morphotypes among *Exophiala* species and other black yeasts, this method is insufficient for definitive identification [57].

#### 6.1.3. PCR and ITS Sequencing

Amplification and sequencing of the internal transcribed spacer (ITS) region of ribosomal DNA remain the gold standard for accurate species-level identification. ITS analysis provides high phylogenetic resolution among black yeasts and is routinely used in clinical practice [110]. Additional targets, such as the D1/D2 domain of the large subunit (26S rDNA), are also used to enhance taxonomic resolution [113,115].

Multigene analyses incorporating ITS and D1/D2 regions have been successfully applied for strain-level differentiation and confirmation of species identity in clinical samples [49,61].

#### 6.1.4. STR Genotyping and AFLP

A short tandem repeat (STR) genotyping scheme developed for *E. dermatitidis* allows for the fine-scale discrimination of isolates [110]. Utilizing six microsatellite loci in a multiplex PCR format confirmed high discriminatory power [110].

Amplified fragment length polymorphism (AFLP) has also been applied to *Exophiala* diagnostics, offering higher genotypic resolution through selective PCR amplification of restriction fragments. When identification is ambiguous, complementary sequencing of loci such as calmodulin or β-tubulin is employed [111].

#### 6.1.5. MALDI-TOF Mass Spectrometry

Matrix-assisted laser desorption ionization time-of-flight mass spectrometry (MALDI-TOF MS) has emerged as a rapid and cost-effective diagnostic tool for fungal pathogens. Historically, its utility in black yeast identification was limited by sparse and inconsistent spectral databases [2]. However, the recent expansion of in-house libraries, particularly those including clinical isolates of *E. dermatitidis*, has improved species-level accuracy [112]. However, although recent examples confirmed the feasibility of reliable identification using MALDI-TOF MS alone [58], most protocols still recommend confirmatory ITS sequencing, especially when clinical implications are significant [62,113].

#### 6.1.6. Diagnostic Challenges and Misidentification

Despite advancements, several diagnostic challenges exist. In a notable case, *E. dermatitidis* was misidentified as *Rhodotorula* by standard laboratory workflows, delaying accurate diagnosis [116,117]. Moreover, standard fungal antigen tests and PCR panels may fail to detect this pathogen, necessitating prolonged culture and morphological confirmation in suspected invasive cases [52]. Also, a major limitation in the routine diagnosis of *E. dermatitidis* remains the absence of targeted PCR assays for formalin-fixed paraffin-embedded (FFPE) samples, a gap that mNGS can partially bridge [114].

### 6.2. Therapeutic Management

The following section synthesizes post-2019 knowledge of therapeutic approaches to *E. dermatitidis*, integrating clinical case reports with in vitro susceptibility data to highlight patterns of drug efficacy, resistance, and treatment outcomes. While no standardized therapeutic guidelines exist, accumulated case reports and in vitro studies help guide decision-making.

#### 6.2.1. Clinical Approaches

Amphotericin B and azole antifungals—particularly itraconazole and voriconazole—are the most frequently used agents in clinical settings. In cases of pulmonary infection, voriconazole has shown good outcomes, with symptom resolution and radiologic improvement after prolonged therapy [113]. Similarly, central line-associated bloodstream infections (CLABSI) have been successfully managed with amphotericin B, voriconazole, and fluconazole, often in combination and alongside catheter removal [62,115].

In pediatric settings and fungemia, combinations such as amphotericin B and flucytosine or azole were common, with mortality rates reaching up to 41.7% depending on underlying conditions [51,115]. Disseminated infections, including CNS involvement, are particularly difficult to treat and associated with poor outcomes even with liposomal amphotericin B and azoles [53].

Corticosteroid use and immunosuppressive therapy have been repeatedly identified as risk factors for *E. dermatitidis* infections and may impair therapeutic efficacy [52]. In mixed infections or biofilm-associated cases, antifungal success often requires addressing co-pathogens and removing infected devices [64].

#### 6.2.2. In Vitro Susceptibility Profiles

Antifungal susceptibility testing consistently reveals a heterogeneous profile for *E. dermatitidis*, with notable resistance to certain agents. These findings are summarized in a Table 6, based on recent studies [10,26,61,64,87,115,118,119].

Recently, it was reported that up to 92% of clinical isolates are capable of forming biofilms [65], which greatly diminish antifungal penetration and efficacy. Biofilm-associated *E. dermatitidis* cells are more resistant to azoles and amphotericin B, necessitating higher drug concentrations and longer treatment durations. Also, further research is needed to clarify the optimal treatment strategies and establish standardized susceptibility thresholds.

#### 6.2.3. Successful Treatment Strategies

Despite the above-mentioned challenges, successful management of *Exophiala dermatitidis* infections has been documented [69,108,115]. However, due to limited knowledge regarding rare fungal pathogens, treatment strategies are typically individualized based on the patient’s clinical condition. For example, in a case of pneumonia, voriconazole was initially recommended and administered; however, adverse effects necessitated a switch to itraconazole [120]. Another successful treatment of a disseminated infection involved a combination of ethanol lock therapy and surgical removal of skin lesions [69]. Although several cases of patient recovery have been reported, the low number of case reports for each type of infection makes it difficult to propose a standardized treatment strategy.

## 7. Conclusions and Future Research

*E. dermatitidis* is increasingly recognized as an opportunistic pathogen with remarkable ecological flexibility and clinical relevance. The recent literature has expanded our understanding of its pathogenic mechanisms, highlighting melanin production, metabolic adaptability, and enzyme-mediated tissue invasion as critical virulence traits. The clinical spectrum has also broadened—from localized infections to severe systemic disease—often occurring in immunocompromised or genetically predisposed hosts.

Advances in molecular diagnostics and proteomics have improved species-level identification, yet challenges remain, particularly in polymicrobial contexts or resource-limited settings. Therapeutically, the lack of standardized guidelines continues to complicate management. Azole antifungals, especially voriconazole and itraconazole, remain the most consistently effective agents, though biofilm formation and variable susceptibility patterns demand individualized treatment approaches.

Despite recent advances, significant gaps remain in our mechanistic understanding of *Exophiala dermatitidis* virulence, immune evasion strategies, and host–pathogen interactions. In particular, the roles of melanin production, thermotolerance, and biofilm formation in disease progression are not yet fully elucidated. Continued research—especially using in vivo models and translational approaches—is essential to better define pathogenic mechanisms, validate potential diagnostic biomarkers, and improve the timeliness and accuracy of clinical diagnosis.

Furthermore, treatment remains challenging due to variable antifungal susceptibility and emerging resistance to first-line agents such as azoles. Future studies should prioritize comparative efficacy trials of existing antifungal regimens, pharmacokinetic/pharmacodynamic profiling in different patient populations (e.g., immunocompromised, pediatric), and the development of antifungal combination therapies. In addition, novel therapeutic strategies—such as immunomodulatory agents, phage-derived enzymes, or antifungal peptides—deserve investigation as potential alternatives to overcome resistance and enhance host response.

Addressing these gaps is critical to reducing the high morbidity and mortality associated with invasive *E. dermatitidis* infections and to preparing for its potential emergence as a more prominent opportunistic pathogen in the era of expanding immunosuppression and climate-driven changes in fungal ecology.

## Figures and Tables

**Table 1:** Overview of recent clinical presentations of *Exophiala dermatitidis*, organized by infection site and host context. The table highlights key pathological features, predisposing conditions, and representative case reports published since 2019.

Clinical Manifestation	Immunocompetence	Predisposing Factors	Outcome	Refs.
Pulmonary (non-CF)	No	Transplant, chemotherapy	Often fatal	[47,48]
CNS	Often yes	CARD9 ^1^, East Asia	High mortality	[49,50]
Fungemia	No	Catheter, malignancy	Poor if delayed	[51,52,53]
Crohn’s disease	Possibly	Immune dysregulation	Variable	[45,54,55,56]
Ocular	No	Surgery, steroids	Graft failure	[57,58,59]
Hair loss/scalp	Possibly	Possibly dysbiosis	Not confirmed	[60]
Polymicrobial (lung, blood)	Both	Biofilm, devices	Complex	[43,61,62,63,64,65,66,67]

^1^ Association with CARD9 deficiency is speculative. CF—cystic fibrosis; CNS—central nervous system.

**Table 2:** Overview of melanin types produced by *Exophiala dermatitidis*, including their biosynthetic pathways, environmental triggers, and hypothesized pathogenic functions.

Melanin Type	Biosynthetic Pathway	Environmental Triggers	Functions	Refs.
DHN–melanin	Polyketide pathway (via Pks1p)	Constitutively produced	Invasion, resistance to ROS, structural reinforcement	[77,78,79]
L-DOPA–melanin	From L-DOPA via laccase/tyrosinase	Host-like conditions (e.g., CNS)	Possible neurotropism mimicry	[76]
Pyomelanin	Tyrosine degradation (via HGA)	Oxidative stress	Iron scavenging, stress resistance	[76]

DHN—1,8-dihydroxynaphthalene; L-DOPA—L-3,4-dihydroxyphenylalanine; Pks1p—polyketide synthase 1 protein; HGA—homogentisic acid; CNS—central nervous system; ROS—reactive oxygen species.

**Table 3:** Summary of enzymatic activities reported in *Exophiala dermatitidis* relevant to its potential virulence.

Enzyme	Function	Detection Frequency	Comments	Refs.
Catalase	Detoxifies H_2_O_2_; oxidative stress protection	100% of strains	Core survival factor; bifunctional catalase/peroxidase also predicted	[84,85,86,87]
Urease	pH neutralization; evasion of macrophages	Variable (5–100%)	Correlates with ammonia tolerance; possibly host-induced	[85,86,87,88,89]
DNase	Degrades NETs/DNA traps	Rare (0–2%) in vitro; present in ex vivo RNA-seq	Likely conditionally expressed	[77,86,87,90]
Protease	Host tissue degradation; immune evasion	Rare in vitro	Serine protease expression upregulated in host context	[77,85,86,91]
Hemolysin	Iron acquisition via RBC lysis	Variable (0–93%)	Only α-hemolysis reported; strain-dependent	[10,85,92]
Phospholipase	Membrane degradation	Not detected	No activity observed under current methods	[86,87,93,94]
Oxidases	ROS metabolism	Not detected	No activity observed under current methods	[95,96,97]
Esterase	Various (e.g., invasion, ROS defense)	Unknown or unexplored	Identified in other fungi; not studied in *E. dermatitidis*	[95,96,97]
SOD				
Hyaluronidase				
Elastase				

SOD—superoxide dismutase; NETs—neutrophil extracellular traps; RBC—red blood cells; ROS—reactive oxygen species.

**Table 4:** Notable examples of clinically relevant carbon sources assimilated by *Exophiala dermatitidis*, and their physiological context in the human host.

Carbon Source	Physiological Context in the Human Host	Refs.
**Common sugars and derivatives**		
Glucose	Abundant in blood and tissues; primary carbon source efficiently assimilated.	[50,86,104]
Fructose	Present in diet and bloodstream at low levels; assimilation likely via sorbitol intermediates.	[10,86,105]
Sucrose	Dietary disaccharide not naturally present in tissues; transiently available in GI tract.	[10,86,104]
Inulin	Dietary fructan polysaccharide not digested by humans; fermented by gut microbiota.	[10,104]
Galactose	Released from mucins and glycoproteins during tissue degradation.	[10,86,104]
N-acetyl-D-glucosamine	Component of microbial and fungal cell walls present in human niches.	[10,86]
**Polyols and sugar alcohols**		
Sorbitol	Accumulates in diabetic tissues as polyol pathway intermediate.	[10,86]
Glycerol	Lipid metabolite, available in tissues and blood.	[10,86,104]
**Organic acids and metabolites**		
2-keto-D-gluconate	Intermediate metabolite in different metabolic pathways.	[86,104]
Succinate	Common TCA cycle intermediate found in host cells and tissues.	[10,104]
Glucuronate	Involved in detoxification pathways; present in extracellular matrix.	[10,104]
Lactate	Present in inflamed tissues and vaginal environments.	[104]
**Neurotransmitters and AAs**		
GABA	Major neurotransmitter in CNS.	[50]
Dopamine	Neurotransmitter and melanin precursor.	[10,50]
Serotonin	CNS and GI tract neurotransmitter.	[10]
Norepinephrine/epinephrine	Neurotransmitters and hormones, present in blood and various tissues.	[10]
Tryptophan	Aromatic amino acid with catabolites linked to virulence.	[10,106]
Glutamate	Major excitatory neurotransmitter in the CNS and a key amino acid in tissue metabolism.	[10,50]
**Aromatic and environmental compounds**		
Phenol	Environmental and host-derived toxin.	[10]
Catechol	Oxidized metabolite and melanin precursor.	[10]

AAs—amino acids; GABA—γ-aminobutyric acid; CNS—central nervous system; GI—gastrointestinal; TCA—tricarboxylic acid cycle.

**Table 5:** Comparative overview of diagnostic methods for *Exophiala dermatitidis*.

Method	Time	Specificity	Advantages	Limitations	Refs.
Culture	2–7 days	Low–Moderate	Widely available, supports further testing	Slow growth, risk of contamination	[2,57,59]
Microscopy	<1 day	Low	Simple, quick	Low discriminatory power	[57]
ITS Sequencing	1–3 days	High	Species-level ID, phylogenetic value	Requires sequencing facility	[48,55,107,108,109]
STR Genotyping	2–3 days	High	Strain typing, outbreak tracing	Requires multiplex PCR and interpretation	[110]
AFLP	2–4 days	High	High genotypic resolution	Technically demanding	[111]
MALDI-TOF MS	<1 day	Moderate–High	Rapid, cost-effective	Database-dependent, variable reliability	[58,62,112,113]
mNGS	1–3 days	High	Unbiased detection of rare/novel pathogens; works on FFPE	Expensive; requires bioinformatics infrastructure	[114]

ITS—internal transcribed spacer; STR—short tandem repeat; AFLP—amplified fragment length polymorphism; MALDI-TOF—matrix-assisted laser desorption ionization time-of-flight; MS—mass spectrometry; mNGS—metagenomic next-generation sequencing; ID—identification; FFPE—formalin-fixed paraffin-embedded; PCR—polymerase chain reaction.

**Table 6:** In vitro efficacy and clinical relevance of antifungal agents against *Exophiala dermatitidis*.

Agent	MIC Range (µg/L)	Median In Vitro Activity	Biofilm Efficacy	Clinical Notes	Refs.
Voriconazole	0.002–8	High	Reduced	First-line therapy in many case reports.	[26,61,87,115,118,119]
Fluconazole	0.5–256	Poor	Poor	Largely ineffective.	[10,26,61,87,115,118,119]
Itraconazole	0.03–2	High	Reduced	Effective in CF and localized infections.	[10,61,64,87,115,118,119]
Posaconazole	0.002–0.5	High	Reduced	Alternative to voriconazole.	[61,87,115,118,119]
Miconazole	0.12–5	Moderate	NI	Rarely used.	[61,118]
Amphotericin B	0.064–2	Moderate	Reduced	Used in severe and disseminated cases.	[26,61,87,115,118]
5-fluorocytosine	1–128	Poor	Poor	Largely ineffective.	[61,87,115]
Micafungin	0.125–16	Poor	Poor	Echinocandin class; limited activity.	[115,118]
Anidulafungin	0.008–32	Poor	Poor	Similar limitations as micafungin.	[26,87,115,119]
Caspofungin	0.008–32	Poor	Poor	Not recommended due to resistance.	[87,115,118,119]
Terbinafine	0.06–0.13	High	High	Rarely used.	[10,115,118]

MIC—minimal inhibitory concentration; NI—not investigated; CF—cystic fibrosis.

## Abbreviations

The following abbreviations are used in this manuscript:

AA	amino acid
AFLP	amplified fragment length polymorphism
AIDS	acquired immunodeficiency syndrome
BCSA	*Burkholderia cepacia* selective agar
BYF	black yeast-like fungi
CARD9	caspase recruitment domain-containing protein 9
CF	cystic fibrosis
CFTR	cystic fibrosis transmembrane regulator
CD	Crohn’s disease
CLABSI	central line-associated bloodstream infections
CLRs	C-type lectin receptors
CNS	central nervous system
COPD	chronic obstructive pulmonary disease
COVID	coronavirus disease
CXCL/R	C-X-C motif chemokine ligand/receptor
D1/D2	domains 1 and 2 of the large subunit ribosomal RNA gene
DHN	1,8-dihydroxynaphthalene
DIC	differential interference contrast
ECA	erythritol chloramphenicol agar
FFPE	formalin-fixed paraffin-embedded
GABA	γ-aminobutyric acid
GI	gastrointestinal
HGA	homogentisic acid
HIV	human immunodeficiency virus
ID	identification
IL	interleukin
INDEL	insertion-deletion
ITS	internal transcribed spacer
L-DOPA	L-3,4-dihydroxyphenylalanine
KS	keto-synthase
MALDI-TOF	matrix-assisted laser desorption ionization time-of-flight
MAPK	mitogen-activated protein kinase
MEA	malt extract agar
MIC	minimal inhibitory concentration
mNGS	metagenomic next-generation sequencing
MS	mass spectrometry
Myb-like	myeloblastosis-like
NET	neutrophil extracellular trap
NF-κB	nuclear factor kappa-light-chain-enhancer of activated B cells
NI	not investigated
PAMP	pathogen-associated molecular pattern
PCR	polymerase chain reaction
Pks	polyketide synthase
PRRs	pattern recognition receptors
RBC	red blood cells
ROS	reactive oxygen species
SDA	Sabouraud dextrose agar
SGCA	Sabouraud gentamicin chloramphenicol agar
SH-SY5Y	human-derived neuroblastoma cell line, subclone of SK-N-SH
SNP	single nucleotide polymorphism
SOD	superoxide dismutase
STR	short tandem repeat
TCA	tricarboxylic acid cycle
T_H_17/22	T helper 17/22
TLRs	Toll-like receptors
TNF-α	tumor necrosis factor α
YPD	yeast extract-peptone-dextrose

## Appendix A. Extended Data on *Exophiala dermatitidis*

### Appendix A.1. Additional Background Information

Phylogenetic analyses based on rDNA sequences have demonstrated a close relationship between *Exophiala* species and their teleomorph *Capronia mansonii*, suggesting they represent different morphs of the same biological species [121].

Other clinically relevant species include: *E. phaeomuriformis*, *E. jeanselmei*, *E. xenobiotica*, *E. lecanii-corni*, *E. bergeri*, *E. mesophila*, *E. campbellii*, *E. spinifera*, and *E. oligosperma* [2,3,4,5,6,7,8].

Although species within the genus *Exophiala* share general morphological characteristics, several display distinct phenotypic and physiological traits that support species-level differentiation. For example, *Exophiala jeanselmei*, a widely distributed pathogen associated with phaeohyphomycosis and mycetoma, was reclassified several years ago as part of a species complex that includes *E. oligosperma*, *E. nishimurae*, and *E. xenobiotica* [122]. These species demonstrate the ability to grow at 37 °C but not at 40 °C, a feature useful in diagnostic settings [122]. In contrast, *E. dermatitidis* demonstrates a polyextremotolerant phenotype, characterized by robust growth at temperatures exceeding 40 °C and adaptation to diverse environmental stressors [2].

### Appendix A.2. Macro- and Micromorphology of Exophiala dermatitidis

Colonies of *Exophiala* spp. appear dark olive-brown to black, and are often olive-gray on the reverse side [123]. Initially, they exhibit a moist, yeast-like, mucilaginous surface with a glossy appearance, which gradually transitions into a pasty, suede-like, or fluffy texture due to the development of aerial gray hyphae. Mature colonies are typically brown to olivaceous-black on the surface, with a black reverse side [123]. Colonies are convex, non-invasive to the substrate, and display a defined mycelial margin.

In early culture stages, round budding yeast-like cells (blastoconidia) appear, often forming long chains. As the colony matures, phaeoid (brown-pigmented), septate hyphae develop, composed of barrel-shaped cells [80]. Conidiogenesis is typically annellidic, involving tube- or rocket-shaped annellides that taper to a long tip and give rise to ellipsoid, unicellular conidia (1–3 × 3–6 µm), typically clustered at the tips or sides of the conidiogenous cells [80].

At ambient temperatures, *E. dermatitidis* exhibits filamentous growth, whereas at 37 °C, it predominantly forms yeast-like cells. Environmental calcium concentration has also been shown to modulate morphogenesis, promoting either hyphal or yeast-like growth depending on availability [2].
